# Supplementary figures and images for: Survival and spread of engineered Mycobacterium smegmatis and associated mycobacteriophage in soil microcosms
Source: Appl Environ Microbiol. 2025 May 21;91(6):e00212-25. doi: 10.1128/aem.00212-25 (PMC12175536; doi:10.1128/aem.00212-25)

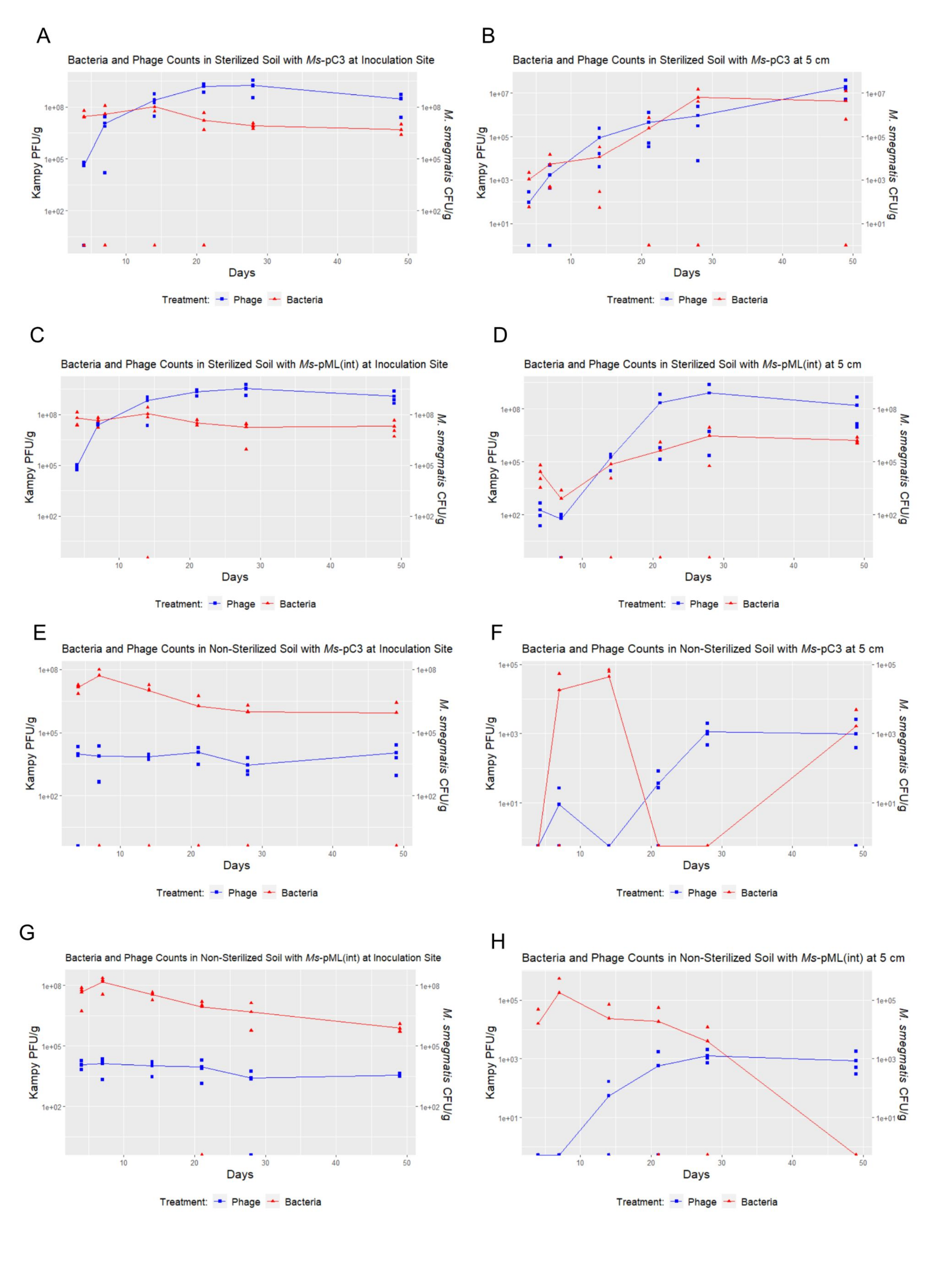

Supplement: Fig. S1 — Comparison of bacteria and phage abundance within each microcosm. [file aem.00212-25-s0001.tiff]
